# Endosurgical Remodeling of Wide-Necked Bifurcation Aneurysms

**DOI:** 10.3389/fneur.2019.00245

**Published:** 2019-03-20

**Authors:** Samantha Dayawansa, Suraj Sulhan, Jason H. Huang, Patrick T. Noonan

**Affiliations:** ^1^Department of Neurosurgery, Baylor Scott & White Health Neuroscience Institute, Temple, TX, United States; ^2^College of Medicine, Texas A&M University, Temple, TX, United States; ^3^Neuroendovascular Surgery, Department of Neurosciences, Doctors Hospital at Renaissance, Edinburg, TX, United States

**Keywords:** wide-necked aneurysms, bifurcation aneurysms, endosurgical remodeling, micro-scaffolding, coil embolization

## Abstract

**Background:** Wide-necked cerebral aneurysms at a bifurcation can be difficult to treat with endovascular techniques despite recent advancements.

**Objective:** We describe a new technique of micro-scaffold remodeling of the aneurysm neck of wide-necked bifurcation aneurysms by placing one or more microcatheters and/or wires in the efferent vessels. We hypothesize that this technique would be a better choice to change the branch angulation, allowing for an improved configuration to stably deploy coils. We present a retrospective case series to illustrate this technique.

**Methods:** 17 wide-necked bifurcation aneurysms in 17 patients were coil embolized using this technique during a 3 year study period. Branch-vessel microcatheters and/or microwires were used to remodel the aneurysm neck and support the coil mass. Statistical analysis of the branch angulation and neck-width changes were performed during treatment. Long-term clinical outcome and follow-up angiography was obtained in 8 patients.

**Results:** Eleven patients had complete occlusion of their aneurysm (Raymond-Roy Class I), and 6 patients had Raymond-Roy Class 2 immediately after treatment. Efferent vessels demonstrated a statistically significant change in angulation with insertion of microcatheters or microwires, while neck width did not change significantly. There were four intraoperative complications and no neurological morbidity in the immediate post-operative period. Complete occlusion was documented for all 10 subjects with long-term follow-up.

**Conclusions:** The micro-scaffold endosurgical remodeling technique is a useful adjunct in treating wide-necked bifurcation aneurysms. By elevating branch vessels away from the aneurysm neck, this technique allows for dense coil packing while decreasing the need for balloon or stent assistance.

## Introduction

The success of coil-embolization in the treatment of intracranial aneurysms is dependent on its morphology, and is challenging in the case of wide-necked aneurysms that are located at a vessel bifurcation (1–3). This is due in part to herniation of coils that subsequently occlude nearby vasculature (4–6). Certain techniques, such as stent-assisted coiling, balloon-assisted coiling, and flow diverters, have been presented in literature as potential avenues for wide-necked aneurysm management (4, 7, 8). Stent-assisted coiling can be challenging at vessel bifurcations necessitating advanced techniques, such as Y-stenting (9). In addition, smaller efferent branches may make stent deployment difficult (10–14). Balloon-assisted coiling is a versatile technique but is associated with serious complications and has a steep-learning curve (6). Dedicated endovascular devices for bifurcation aneurysms such as the PulseRider Aneurysm Neck Reconstruction Device, which carries and HDE approval in the United States, and intra-saccular devices such as the Woven Endovascular Bridge (WEB) have been engineered to facilitate coil embolization of bifurcation aneurysms (15, 16).

In an era when dedicated bifurcation aneurysm devices were not available, we devised a technique to address the challenges of treating wide-necked, bifurcation aneurysms for which available reported techniques were deemed to be either unfeasible or insufficient. Multiple microcatheters and/or microwires were used to temporarily change the configuration of the aneurysm neck and the bifurcation branches. We hypothesized that using these adjunctive devices to change the angle of the branches would provide a micro-scaffold with which to introduce and safely deploy coils. After the deployment of coils and removal of microcatheters and/or wires, the angle of the scaffolded branches reverts to its pre-procedural configuration, allowing the coils to remain in place thus obliterating the aneurysm.

## Materials and Methods

Between May 2011 and September 2014, we identified 17 wide-necked, bifurcation aneurysms in 17 patients at our institution that were treated with our technique. The clinical characteristics of these patients are included in Table 1. This figure includes detailed information about each patient, including their clinical presentation, location and size of the aneurysm, neck width, efferent vessel angle change, and complications. Representative vessel angles before, during and after the procedure were also recorded. Statistical analysis of the angles before, during and after the procedure were analyzed by running a paired sample *t*-test. This was done with the assistance of statistical software Statistica (V.7). The number of coils and the degree of occlusion (Raymond-Roy Class) were analyzed. Follow-up catheter-based angiography was available in 10 patients over a 6–28 month period. Follow-up magnetic resonance angiography (MRA) was available in an additional 4 patients over a 2–24 month period (See Supplemental Figure 1).

**Table 1:** Clinical characteristics of wide-necked bifurcation aneurysms.

**Subject#**	**Gender**	**Age (at procedure)**	**SAH on presentation**	**Aneurysm location**	**Aneurysm dimensions (mm)**	**Neck width (mm)**	**# Coils**	**Efferent vessel angle change (^**°**^)**	**Complications**	**Outcome**	**Occlusion**	**LOS (days)**	**Follow-up modality and outcome**
1	M	79	Unruptured	L A1A2ACoA	7 × 4.5 × 6	3.95	12	51.8	compromised L A2 flow resolved post intra-arterial NTG	L ICA and R PCA stenoses	Complete	2	MRA (12mo): complete occlusion
2	M	77	Unruptured	R MCA	7 × 6 × 4	2	10	43.3	None	No compromise of parent or branch vessels.	Complete	1	MRA (12mo): complete occlusion
3	F	69	Unruptured	L MCA	9.2 × 8.2 × 6	7.25	12	36.9	None	60% stenosis of L M2 branch	>95%	1	Angiography (10mo): complete occlusion
4	F	55	Unruptured	Basilar terminus	9.7 × 8 × 7.6	7	9	39.8	None	No compromise of parent or branch vessels.	Complete	1	Angiography (27mo): complete occlusion
5	F	55	Unruptured	Basilar terminus	11 × 8 × 9	4.68	13	46.3	None	No compromise of parent or branch vessels.	>95%	1	Angiography (26mo): complete occlusion
6	F	50	Unruptured	Basilar terminus	4 × 3.8 × 5.8	2.95	5	23.1	None	No compromise of parent or branch vessels.	Complete	1	MRA (2mo): complete occlusion
7	F	61	Unruptured	R MCA	7 × 10 × 6.2	3.77	5	42.2	mild diffuse SAH in high R sylvian cistern	Patency of parent and vessel branches	Complete	5	Angiography (27mo): complete occlusion
8	F	55	Unruptured	R MCA	12.5 × 15.6 × 14	6.28	21	37.3	None	Patency of parent and vessel branches, patency of aneurysm neck giving rise to MCA branches	>95%	1	Angiography (6mo): complete occlusion; mild stent stenosis
9	M	56	Unruptured	RA1A2ACoA	6.5 × 5.5 × 3.7	4.43	9	39.4	None	Change in dominance of the A1 segments noted after embolization. No compromise of parent or branch vessels.	Complete	1	No follow-up imaging
10	F	62	Unruptured	R MCA	8 × 5 × 6	7.62	4	41.1	Brief cessation of antegrade flow in nondominant superior division trunk resolved with removal of coil	Patency of parent and vessel branches; slowed antegrade flow in nondominant MCA superior division trunk; Contrast extravasation in R sylvian cistern with stable SAH on TCD	Complete	3	Angiography (28mo): complete occlusion; mild stent stenosis; 60% stenosis of R MCA
11	F	58	Unruptured	R MCA	7 × 10.10 × 9.7	5.2	16	31.8	None	No compromise of parent or branch vessels.	Complete	1	No follow-up imaging.
12	M	38	Unruptured	L MCA	4.2 × 4.1 × 7.8	3.5	2	35.7	None	No compromise of parent or branch vessels.	>90%	1	Angiography (10mo): complete occlusion
13	M	59	Ruptured	ACoA	8 × 6 × 12	4.76	2	46.7	Mild impairment of cross-filling via the ACoA due to slight coil prolapse	Slight delayed flow in L A2	Complete	14	No follow-up imaging
14	F	71	Ruptured	LA1A2ACoA	9.2 × 9.8 × 12.8	4.44	11	25.8	None	No compromise of parent or branch vessels.	Complete	16	No follow-up imaging
15	F	47	Ruptured	L MCA	6 × 5.4 × 4.1	3.84	3	48.9	None	No compromise of parent or branch vessels.	>90%	17	No follow-up imaging
16	F	51	Unruptured	R MCA	6.2 × 6.4 × 7.7	4.72	7	22.2	None	No compromise of parent or branch vessels.	>95%	1	Angiography (6mo): near complete occlusion
17	F	62	Unruptured	L ICA	6 × 5 × 5	5.01	6	14.1	None	No compromise of parent or branch vessels.	Complete	3	MRA (24mo): complete occlusion

*Clinical details and outcomes of 17 bifurcation aneurysms in 17 patients using the described technique–bifurcation remodeling with microwires and microcatheters followed by endovascular coiling. A1, Anterior Cerebral Artery, First Segment; A2, Anterior Cerebral Artery, Second Segment; ACoA, Anterior Communicating Artery; ICA, Internal Carotid Artery; LOS, Length of Stay; MCA, Middle Cerebral Artery; MRA, Magnetic Resonance Angiography; NTG, Nitroglycerin; PCA, Posterior Cerebral Artery; SAH, Subarachnoid Hemorrhage; TCD, Transcranial Doppler*.

## Coil Embolization Technique

The configuration of the aneurysm neck, as well as its relationship to the parent and branch vessels, was analyzed using multiplanar 2D and 3D reconstructions derived from computed tomographic (CT) angiograms prior to procedure. All patients were coil-embolized under general anesthesia using a bi-plane Siemens Neurostar (Siemens Medical Solutions, Malvern, PA). Systemic heparin anticoagulation was used and monitored appropriately in all cases. In order to change the configuration of the bifurcation and the aneurysm ostium, a combination of up to 3 microcatheters (Excelsior SL-10, Stryker Neurovascular, Fremont CA, USA) and/or microwires (Synchro 2, Stryker Neurovascular) were placed in the bifurcating vasculature at the aneurysm. Change in the bifurcation configuration was confirmed by roadmap fluoroscopy. All catheters were set up with continuous heparinized saline flush (4,000 u/liter).

When it was anticipated that coil loops in the aneurysm might obstruct the origin of a perforatoring vessel, a microcatheter was placed in the vessel with or without a microwire. The radiopaque wire provided a marker to select a working view during aneurysm coiling that demonstrated separation of the coil mass above from the radiopaque wire below. Because the wire tended to bow toward the aneurysm neck, the space below the wire relative to the aneurysm neck could be assured to be a patent branch. This was confirmed by periodic angiography; even when a large coil mass would have otherwise obscured the branch origin.

All patients required only a single femoral artery access. In most the cases, a 6 Fr × 90 cm sheath (Pinnacle, Terumo, Somerset NJ, and Neuron Max, Penumbra, Alameda CA) was used to accommodate all microwires and microcatheters. In the remainder of cases, a 6 Fr guide catheter (Envoy, Codman Neuro, Raynham MA) was used. The microcatheters and microwires were inserted at the sheath or guide catheter hub via stacked rotating hemostatic valves. By using an exchange microwire, a microcatheter which had been used to selectively catheterize a branch vessel could be removed and reused to selectively catheterize the aneurysm leaving only the exchange microwire in place in the branch. In other cases, microcatheteters with or without contained standard length microwires were left in branches and the microcatheters were continuously flushed. The presence of a microwire in a microcatheter tended to produce a greater change in branch vessel angulation than a microcatheter alone. Only the stented patients received adjunctive dual agent antiplatelet therapy. After temporarily reconfiguration of the bifurcation had been achieved using adjunctive microcatheters and/or microwires, up to 2 microcatheters were placed in the target aneurysm. Detachable coils (Axium, Medtronic Neurovascular, or Target, Stryker Neurovascular) were subsequently used to embolize the aneurysm.

During the course of target bifurcation aneurysm treatment in 3 patients, adjunctive placement of a stent (Neuroform EZ, Stryker Neurovascular) was necessary. In 2 of these cases, stent implantation in the dominant bifurcation branch having a lumen diameter ≥ 2 mm was anticipated to leave a portion of the aneurysm neck uncovered. Because the remaining < 2 mm diameter branch arising from the uncovered portion of the aneurysm neck supplied eloquent territory, symptomatic branch occlusion as a result of coiling was anticipated unless a method to preserve branch patency was applied. In each case, the nondominant branch and its eloquent territory was satisfactorily protected by temporary placement of a microcatheter containing a microwire. The microcatheter containing the microwire in the branch was jailed by the stent, and the microcatheter was easily removed after completion of coiling. In the last of these cases, a 4.5 mm diameter stent was used as a waffle cone to protect the central neck of an MCA bifurcation aneurysm with a neck that was wider than the expanded dimension of the stent and from which small diameter bifurcation branches arising at the lateral neck margins could not be covered by the stent in the central neck. In that case, microwire containing microcatheters were placed in each of the MCA bifurcation branches prior to placing the stent in order to provide coil support in the portions of the aneurysm neck that could not be covered by the stent. This was also done to mark the positions of the branch origins with the radiopaque microwires, and to preserve branch patency during the coiling procedure. As in the first two cases, the microcatheters were easily retrieved from the branch vessels without displacement of the stent or occlusion of the branch vessels.

## Results

This study includes 17 aneurysms with findings as reported in Table 1. Remodeling of the aneurysm neck was confirmed in all cases as indicated by a change in branch vessel angulation during the procedure. A minimum of 2 coils to a maximum of 21 coils were placed. Immediately after the procedure, total radiographic, Raymond Roy Class 1, occlusion was achieved in 11 aneurysms with the remaining 6 aneurysms achieving Raymond Roy Class 2 occlusion. Nine aneurysms were located at the MCA bifurcation, four at the anterior communicating artery complex (ACoA), three at the basilar apex, and one at the ICA bifurcation.

**Figure 1:**
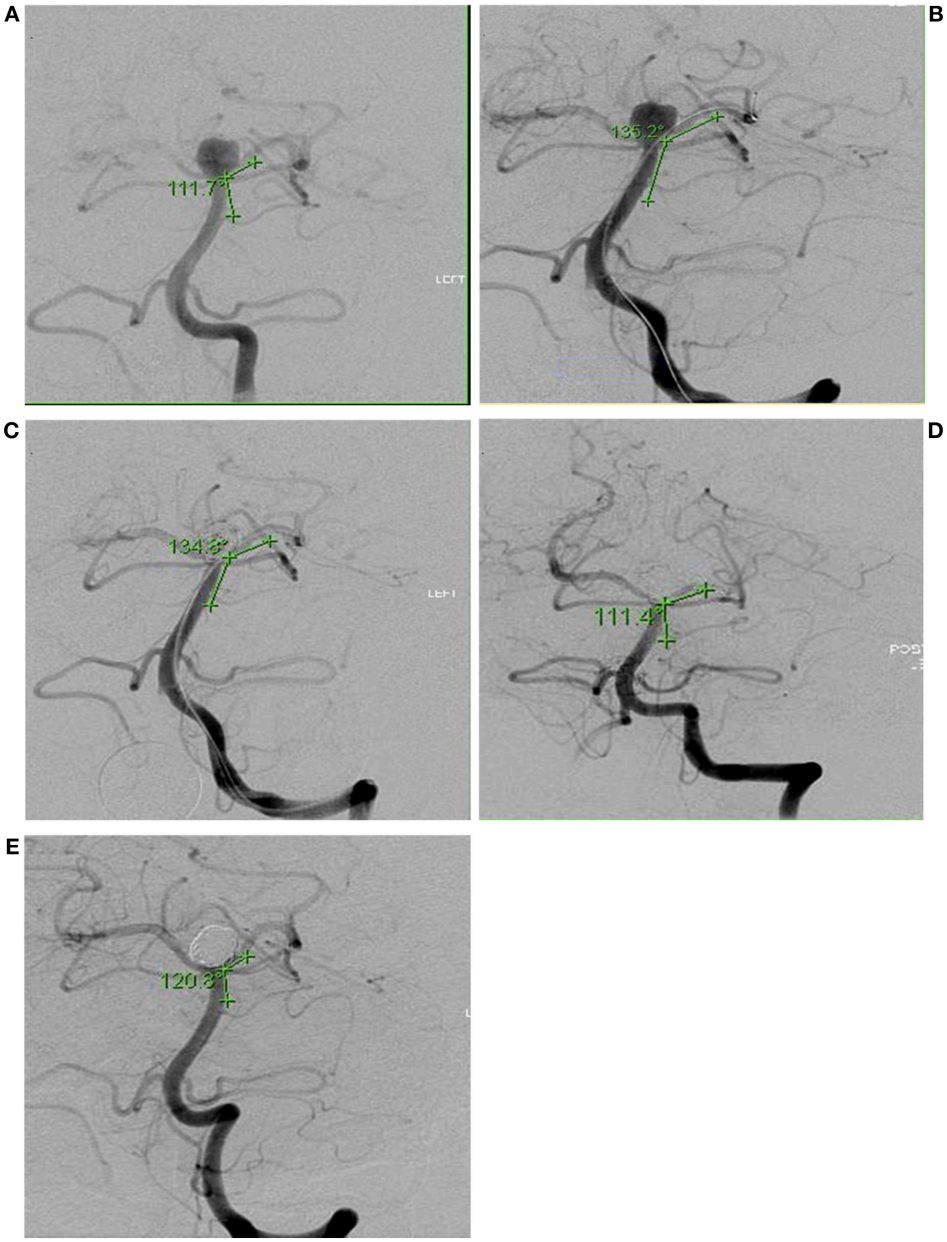
Intra-operative angiography of Subject 6 showing angle of incidence between parent vessel and branch vessel to receive second microcatheter. Angles were measured in 5 conditions: **(A)** Before device insertion (111.7°), **(B)** During microcatheter insertion (135.2°), **(C)** During embolization with microcatheters and coils (134.8°), **(D)** After completion of embolization and removal of all devices (111.4°), **(E)** Follow-up angiography 27 months later (120.8°).

Representative vessel angle before and during the procedure, as well as its normalization afterwards, are shown in Figure 1. Figure 1E was taken during a follow up visit. Vessel trajectories were drawn, as seen in Figure 1, to measure the angle and calculate subsequent change. These trajectories, however, did not always fall along the long axis of the vessels as seen on Figure 1. We measured the angle from several trajectories to confirm our readings were accurate. The final angle was an average of several different measurements.

**Figure 2:**
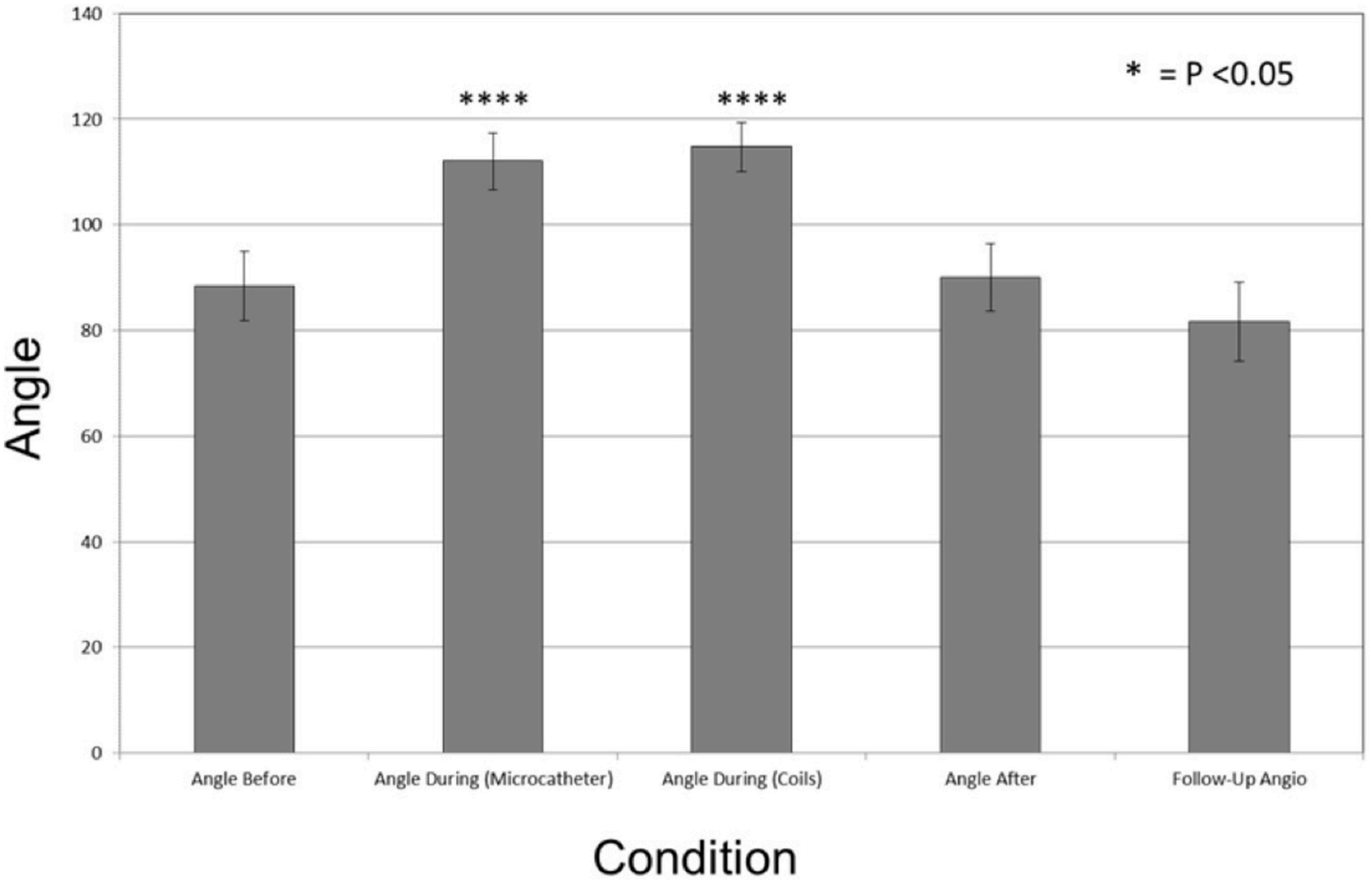
Bar graph plotting the mean degrees of angle change in during the coiling procedure. Angle before (88.42° + 6.59), Angle during (Microcatheter) (112.03° + 5.36), Angle during Coils (114.72° + 4.60), Angle after (90.04° + 6.39), and Follow-Up Angio (81.58° + 7.42). The mean angle change between these conditions was 37° ± 2.5. Vertical line represents Standard Error (SE). during (Microcatheter) and Angles during (Coils) were statistically significant in comparison to the baseline angle before with *p* < 0.001 and *p* < 0.001, respectively, and denoted with ^*^p < 0.05, ^****^p < 0.0001.

The mean angle change before and during the procedure was 37.5° with a standard deviation (±) of 2.5. This represents the degree of conformational change of the aneurysm neck. Branch angles before and after embolization stayed the same. Long-term follow-up angiograms on 10 aneurysms showed no long-term changes in branch angulation. Mean + SE angle changes of all patients are shown in Figure 2. The mean neck width values before, during and after for 17 aneurysms were same (4.92 ± 0.32 mm, 4.92 ± 0.30 mm, 4.96 ± 0.31 mm, respectively). Similar comparisons were made on 8 subjects with follow-up angiograms. There was no significant difference.

## Complications

There were no vessel occlusions and no neurological deficits in the immediate post-operative period. Complications included 2 cases of asymptomatic localized subarachnoid hemorrhage and a thromboembolic event that caused temporary mild hand weakness and mild expressive aphasia. No evidence of frank wire perforation was seen in either case. The localized hemorrhage may have been due to stretching of very small perforators when the angulation of the branch vessels was changed by the neck remodeling wires contained in these branches. An asymptomatic branch vessel stenosis and a symptomatic MCA bifurcation occlusion occurred in late follow-up. The latter event occurred in a patient who received a Neuroform stent (Stryker Neurovascular, Fremont CA) and self-discontinued antiplatelet medication within 3 months of the procedure (subject 8). There was a 10% incidence of thromboembolic events, and a 5% incidence of SAH in the periprocedural period (up to 2 weeks postop). The rate of permanent neurologic deficits in the periprocedural period was 0%. We recognize that the incidence of complications is not zero in our patient group, but is an improvement from conventional endovascular methods and also similar to recent devices reported in literature (17–19).

## Discussion

**Figure 3:**
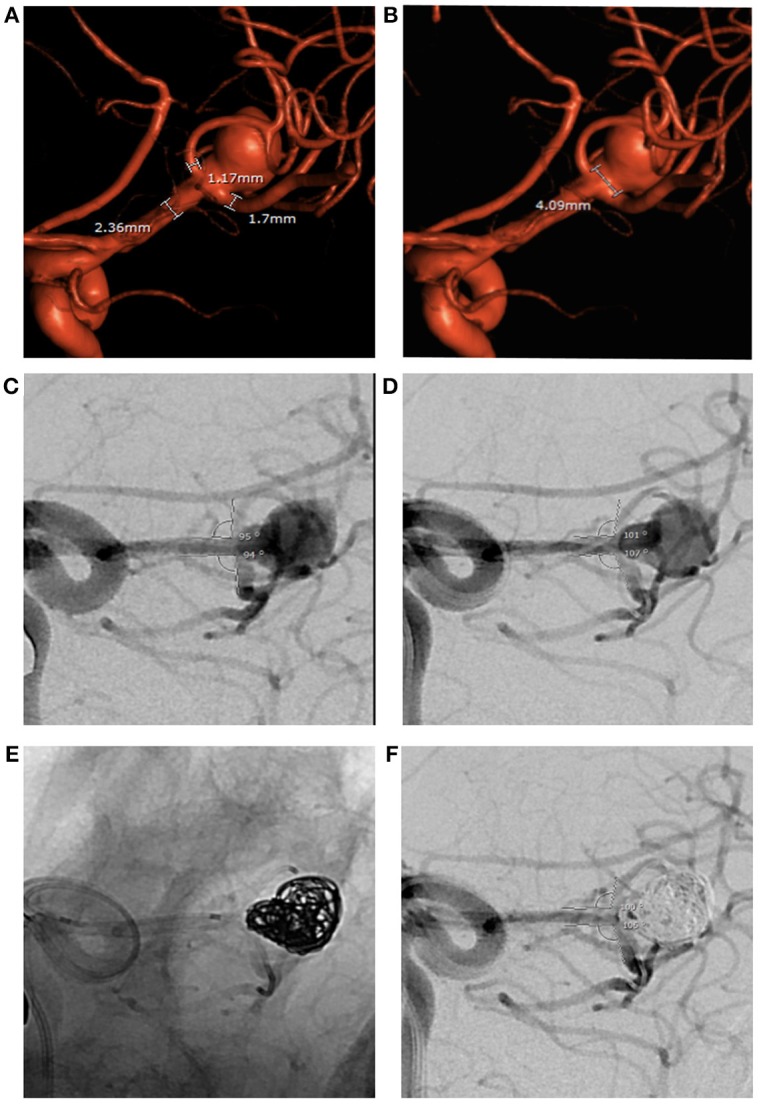
Large ruptured middle cerebral artery bifurcation aneurysm treated by PTN in 2018. **(A,B)** Pre-op 3D angiography: wide aneurysm neck, parent and branch vessel diameters below minimum limits for both PulseRider Aneurysn Neck Reconstruction Device and all HDE stents. **(C,D)** Bifurcation branch angles in working view. **(E,F)** Change in bifurcation branch angles after placement of a microcatheter in each branch persists during aneurysm coiling.

We describe a micro-scaffold technique to assist coil embolization of wide-necked bifurcation aneurysms using microcatheters and/or microwires placed across the aneurysm neck. This induces a conformational change in the bifurcation branch vessels. This temporary mechanical alteration of the vascular geometry often proves sufficient to protect vessel patency during coil embolization, particularly when parent vessel and/or branch vessel lumen diameter is not optimal for stent placement. This method also obviates repeated cycles of balloon inflation and deflation that may compromise vessel wall integrity and protects distal perfused parenchyma at risk of ischemic complications. The presence of these temporary devices, which tend to straighten the branch vessel vasculature and bow toward the aneurysm neck, provides buttressing of the coil mass when it is most needed during formation of a stable coil mass with the initial framing coils. We noted that the use of the devices changed the anatomic conformation of the bifurcation by altering the angle of incidence of the branch vessels relative to the course of the parent vessel. This narrows the angle between the branch vessels, and modifies the geometrical configuration of the aneurysm neck (Figure 3). In some instances, this new configuration allowed for better visualization of eccentric lobes and daughter sacs innate to the aneurysm's anatomy. Additionally, the presence of radiopaque microcatheters and microwires within branch vessels allowed for a precise determination of the position of those vessels relative to the aneurysm and allowed for assessment of their luminal integrity when they would have otherwise been obscured as the aneurysm was filled by coils.

**Figure 4:**
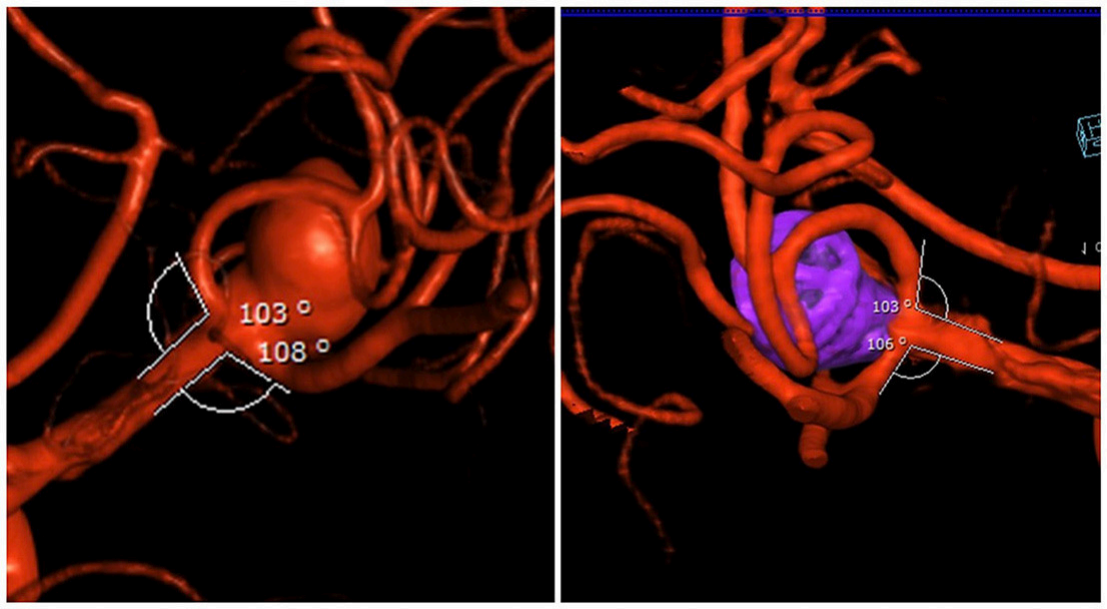
**(Left)** pre-op bifurcation branch angles of the aneurysm. **(Right)** post-op: Raymond 1 occlusion and restoration of native bifurcation branch angles after removal of microcatheters.

We found that the efferent vessel angle changed while the neck width of aneurysm remained the same. The subsequent removal of the microcatheters allowed for reversion of the branches to the initial configuration, allowing for coil retention within the aneurysmal fundus without herniation into parent vessels (Figure 4). This technique demonstrated a Raymond Roy Class 1 or 2 occlusion in all 17 aneurysms initially and total occlusion in all 8 aneurysms with long-term follow-up.

One of the limitations of this study is that multiple measurements from different trajectories could affect consistent measurements. This was one of the areas we spent a significant amount of time measuring and calculating. The angles would indeed change slightly when the trajectories were different; the final angle was an average of different measurements.

Treatment of wide-necked bifurcation aneurysms with coils alone results in recanalization rates of 50–60% and recurrence rates of 30–40% (20). The introduction of balloon-assisted coiling and stent-assisted coiling broadened the spectrum of aneurysms that could be treated endovascularly (21–23). Tahtinen et al showed that stent-assisted coil embolization results in an increased rate of technical success over coils alone (72% in 61 patients with ruptured wide-necked intracranial aneurysms) (24). Balloon remodeling achieves similar rates of complete occlusion (70–80%), with a reported failure rate of 10% and a mortality and morbidity rate of 5%; the most notable risk described was embolic phenomena (25). However, these techniques are associated with complications such as parent vessel occlusion, stenosis resulting from stent placement, and difficulty with optimal balloon positioning (10, 26–28) Y-stenting displayed an increase in immediate occlusion rate (around 87%) and long-term stability, with a late angiographic occlusion rate of 93% and complications documented in only 4% of dual stent cases (29). Both balloon-assisted coiling and stent-assisted coiling reduce the risk of future coil compaction compared to treatment with coils alone (30, 31). Studies of flow-diversion devices report a very low complete angiographic occlusion rate immediately after a procedure and a higher complete occlusion rate of up to 90–95% at 6–12 months of angiographic follow-up. Nonetheless, complete angiographic and volumetric occlusion, compact coiling, and adequate spherical support within aneurysmal sac may still be difficult to achieve (32–34).

Use of the micro-scaffolding technique substituted for an infeasible Y stenting technique in the first 2 of our stented cases, and allowed endovascular treatment of a bifurcation aneurysm having an exceptionally wide neck and small diameter branches while avoiding use of infeasible double Y or X techniques in the last case. Although a stent was implanted in each of these 3 cases, a stent alone was not anticipated to preserve both bifurcation branches, and successful endovascular occlusion of the aneurysm with preservation of branch vessel patency was made possible by concomitant use of aneurysm neck micro-scaffolding technique we have described.

In contradistinction to balloon remodeling techniques, during each of our micro-scaffolding assisted coil embolization procedures parent and branch vessel patency as confirmed by periodic angiography was maintained at all times, neither parent nor branch vessels were subjected to potential trauma caused by repeated cycles of micro-balloon inflation and deflation, and ischemia in downstream parenchyma was avoided.

More recently, devices such as PulseRider Aneurysm Neck Reconstruction Device (Pulsar Vascular, Los Gatos, California) and the Woven EndoBridge (WEB) device (Sequent Medical Inc, Aliso Viejo, California, USA) have been proposed for use in the treatment of bifurcation aneurysms. The PulseRider is a self-expanding implant that is fully retrievable, adaptable stent with an open leaflet structure that uses less metal than a conventional stent, allowing the majority of the device's surface area to cover the neck of the aneurysm (35). The WEB is a densely woven, flow-disrupting device that relies on its shape memory to conform and occlude large wide-necked aneurysms (15). At the time of data collection for this study, neither PulseRider nor WEB were not available for sale in the United States. Both the Waffle technique and the double microcatheter techniques have been described by other authors but reports of their use are limited.

Although these results seem promising, additional experience is needed to confirm the outcomes suggested by our experience using the micro-scaffolding assisted embolization technique in a small number of patients. Our study is also limited the lack of a concurrent control group with which to compare our results. However, we feel that our results compare favorably to published occlusion rates for stent-assisted coiling of wide-necked aneurysms. Notably, some newer devices discussed above received marketing approval on the basis of a small single arm trial (18). Another advantage of the technique we describe is that use of a humanitarian device exemption (HDE) device is usually not necessary. At the time of data collection a PMA approved intracranial stent was not available for sale in the United States. Because the micro-scaffolding technique utilizes 510 K and PMA approved devices within their on-label directions for use (DFU), IRB oversight is only necessary in exceptional cases that may require concomitant use of an HDE device. It is also far less expensive than procedures involving implantation of permanent intraluminal devices, e.g., stents and PulseRider, and it may be used in small caliber vessels (16, 35). Many dedicated permanent implanted intraluminal neck buttressing devices, such as stents and PulseRider, are limited to vessels having a lumen diameter meeting or exceeding a minimum diameter that was verified by animal testing, tested in clinical trials, and stated in the approved labeling. The micro-scaffolding technique we describe was an effective alternative method to protect parent and branch vessels with lumen diameters less than the minimum acceptable for permanent intraluminal implants (36).

We admit that our study is also limited by lack of follow-up in all patients. Further long-term anatomic and clinical follow-up will be needed to identify the subset of aneurysms that will most benefit from use of the micro-scaffolding assisted coil embolization technique and to fully assess the stability of bifurcation aneurysm occlusion achieved by the micro-scaffolding technique we have described.

## Conclusion

Wide-necked, bifurcation aneurysms encompassing branch vessels remain a challenge in endovascular neurosurgery due in part to the risk of compromise of nearby vasculature during coil embolization. Microwires and microcatheters may be used to remodel and reconstruct the local vascular anatomy and enable endosurgical repair. These temporary devices modify the conformation of the bifurcation vessels as evidenced by our pre- and post-embolization angle of incidence measurements as well as the change in the configuration of the aneurysm ostium. Additionally, they permit satisfactory coil embolization while concomitantly protecting parent and branch vessel lumens during exclusion of the aneurysm from the intracranial circulation. By using these simple, relatively inexpensive, and readily available adjunctive devices, the operator may fortify the aneurysm neck, induce local vascular conformational change, and provide a stable micro-scaffold to form an intra-aneurysmal coil mass. Technical challenges associated with balloon-assisted coiling and complex stent-assisted coiling, such as Y-stenting, are avoided; while satisfactory aneurysm occlusion, preserved vessel patency, decreased use of dual-antiplatelet therapy, and a low rate of recanalization requiring retreatment are achieved. The micro-scaffold technique provides a promising adjunctive treatment modality in selected cases.

## Author's Note

The views expressed are those of the author (PN) and do not necessarily reflect the official policy or position of the Food and Drug Administration, the Department of Health and Human Services, or the United States Government.

## Informed Consent

Institutional Review Board approval and patient consents were obtained before data was retrospectively collected.

## Ethics Statement

All procedures performed in studies involving human participants were in accordance with the ethical standards of the institutional and/or national research committee and with the 1964 Helsinki declaration and its later amendments or comparable ethical standards. All devices utilized in the embolization procedures had PMA, 510(k), or HDE approvals and were on the shelf.

## Author Contributions

All authors listed have made a substantial, direct and intellectual contribution to the work, and approved it for publication.

### Conflict of Interest Statement

The authors declare that the research was conducted in the absence of any commercial or financial relationships that could be construed as a potential conflict of interest.

## Supplementary Material

Supplemental Figure 1 **(A,B)** Pre-op angiogram depicting the aneurysm and neck width. **(C,D)** Post-op depicting. Raymond 1 occlusion and restoration of native bifurcation branch angles after removal of microcatheters.
