# Supplementary figures and images for: Endosurgical Remodeling of Wide-Necked Bifurcation Aneurysms
Source: Front Neurol. 2019 Mar 20;10:245. doi: 10.3389/fneur.2019.00245 (PMC6435532; doi:10.3389/fneur.2019.00245)

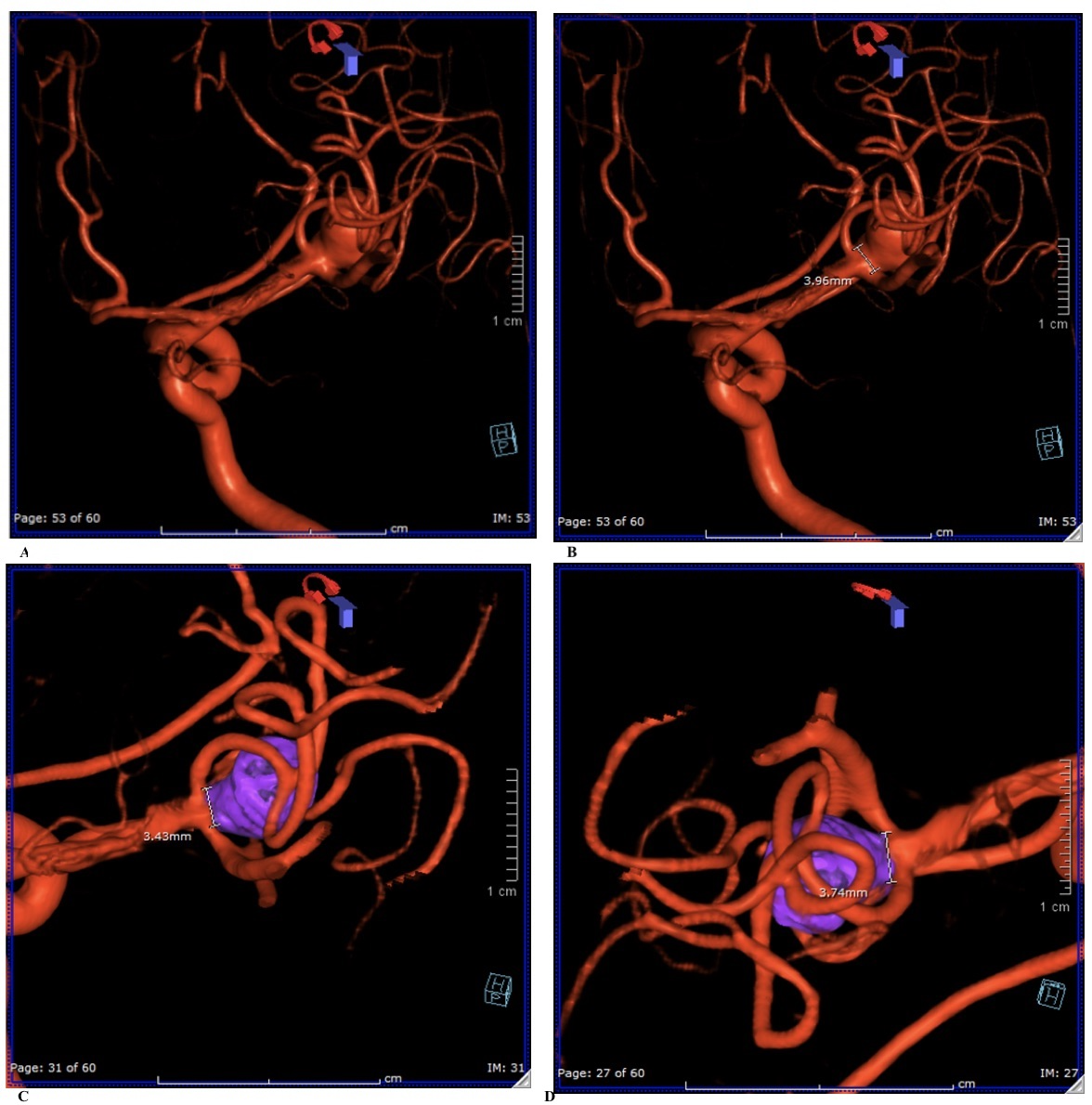

Supplement: Supplemental Figure 1 — (A,B) Pre-op angiogram depicting the aneurysm and neck width. (C,D) Post-op depicting. Raymond 1 occlusion and restoration of native bifurcation branch angles after removal of microcatheters. [file Image_1.TIFF]
